# A Review of Research Conducted with Probiotic* E. coli* Marketed as Symbioflor

**DOI:** 10.1155/2016/3535621

**Published:** 2016-11-22

**Authors:** Claudia Beimfohr

**Affiliations:** Vermicon AG, Emmy-Noether Strasse 2, 80992 München, Germany

## Abstract

This review article summarizes the scientific literature that is currently available about a probiotic* E. coli* that is known under the name Symbioflor* E. coli*. The probiotic is marketed for human use and has been subjected to over 20 years of scientific research. As is presented here, the available literature not only contains multiple works to investigate and analyse the probiotic activity of this* E. coli*, but also describes a variety of other research experiments, dealing with a surprising and interesting range of subjects. By compiling all these works into one review article, more insights into this interesting probiotic* E. coli* were obtained.

## 1. Introduction

Most microbiological reviews focus on a particular scientific subject or technique or summarize the latest research performed with a particular species. This review article follows an alternative approach, as it is focused on a particular type of* Escherichia coli. *The species of* E. coli* is probably the most studied species of all known bacteria, and as a consequence an impressive amount of information is available about it. The species is, however, also very diverse, as it comprises both commensal, benign (probiotic) and pathogenic strains. Naturally, the latter are mostly studied for their pathogenic potential, but a surprising array of methods and investigations have been applied to the commensal and probiotic representatives of this species. The best-known example of the latter is* E. coli *Nissle 1917, which has been subject to a lot of research. The properties of that strain have been reviewed elsewhere [1], but here all peer-reviewed publications dealing with a lesser known probiotic representative of the species are summarized, works performed with Symbioflor* E. coli*. This probiotic product is produced by SymbioPharm GmbH (Herborn, Germany) and is marketed for prevention and treatment of gastrointestinal conditions (e.g., irritable bowel syndrome).

Since Symbioflor* E. coli* is less known than* E. coli *Nissle 1917, some of its characteristics of the commercial product are presented in Table 1. The most obvious difference to probiotic Mutaflor (*E. coli *Nissle 1917) is that Symbioflor contains six genotypes of* E. coli*. The recommended daily dose of Symbioflor is about 100 times lower than that of Mutaflor.

A literature search was conducted in December 2015 to identify the scientific research that has been conducted up to that date with the product or its bacteria and to evaluate what lessons were learned from these works. Some of these studies were commissioned by SymbioPharm, while others were initiated by researchers who decided to use this commercial product, with permission or without contacting the company. Commissioned research is practical and essential, for instance, to investigate the properties and effects of the commercialized bacteria by means of techniques that are only available to the scientific community. From years of experience working with SymbioPharm, this author has the impression that company welcomes the outcome of properly performed scientific work, whether the resulting findings are in line with their commercial interests or not. As long as commissioned work is declared as such (which in scientific publications must be stated in the disclosure of potential conflict of interests) and unfavourable results are not omitted, this practice is completely acceptable from a scientific point of view.

The fact that scientists sometimes decide to use commercial bacterial strains for their experiments can have various reasons, such as a wish to understand if and how the product works or to compare these bacteria with others. It is common, though not required, to ask permission from the commercial producer for such studies; both practices are acceptable. A third type of publications is to combine and compare published findings from multiple authors and present these in the form of a review article, a systematic review, or a meta-analysis. A review article is usually a snapshot of literature findings, in which the authors present the current state of the art, briefly summarize findings from individual publications, and then comment or interpret these findings. In the field of probiotic research, review articles are abundant in comparison to primary literature (i.e., publications describing experimental results). In fact, for two decades, there seemed to be more review articles being published than serious primary literature proving efficacy of probiotics or providing mechanistic explanations of their actions; while multiple review authors tried to convince their colleagues of the functionality of probiotics or lack thereof, the works they selected in their reviews were often biased towards one view or another. The overdose of biased review articles and their frequently subjective views did not help probiotic research to establish a serious position in microbiological science. Fortunately, in the past two decades, this trend was corrected, as more serious, scientifically sound, primary research works are now the norm.

In a systematic review, the included articles are being selected based on predefined criteria, aiming for a more objective coverage of the literature. This is often combined with a meta-analysis of the data, in particular when dealing with clinical studies. Meta-analyses were originally developed to independently evaluate results from clinical studies on medical treatments: by combining results from various clinical trials the effects produced stronger statistical power. A meta-analysis can also identify publication bias, indicating that unfavoured results are being underrepresented. Although the current review is not a systematic review in* sensu stricto*, inclusion criteria were predefined to select works of quality, while literature searches were broadened beyond the initial search terms, in an attempt to cover as many publications as possible.

## 2. Approach

The predefined inclusion selection criteria for publications were as follows.

1. Included papers should deal with Symbioflor or the* E. coli *bacteria; this product contains of parts thereof, described under various names (*E. coli* DSM 17257,* E. coli* G1/2 also known as DSM 16441,* E. coli* G3/10 aka DSM 16443, other genotypes of Symbioflor* E. coli*, and any publications in which alternative names for these bacteria were used). Since in some countries another product is marketed as Symbioflor 1, which contains* Enterococcus* bacteria, where the product of interest is called Symbioflor 2, it was ensured that the identified literature indeed dealt with Symbioflor* E. coli*.
2. Included papers must have been peer-reviewed prior to publication. This applies to most scientific journals and to some books, though in some cases peer-review or lack thereof had to be assumed (see point (4)).

Exclusion criteria were

meeting proceedings and published abstracts of posters or presentations;
contributions to “Arzneiverordnungs-Report,” “HNO-Praxis Heute,” “Arzneimittel-Forschung,” “Facharztwissen HNO-Heilkunde,” and multiauthor books (peer-review status unclear);
case reports and Ph.D. theses;
primary publications in which the product of interest was only mentioned in the introduction or discussion;
genome or sequence comparisons in which the genomes of Symbioflor bacteria were included for comparison only, without revealing novel insights;
review articles and book chapters in which primary papers were cited without adding more information than was described in the citations themselves;
publications in languages other than English or German, since their content could not be assessed.

Searches were performed at first using PubMed and then using Google Scholar. For all relevant papers retrieved in PubMed, the links that this database provides to “related articles” and “cited by” were followed to identify other works of interest. The searches in PubMed were performed to saturation, meaning all retrieved hits with the search terms “Symbioflor 2,” “*E. coli* DSM 17257,” and “*E. coli* G1/2” were checked, although “related articles” links were not followed to completion for any of these. In Google Scholar, the first 300 hits obtained with the search term “Symbioflor 2” were screened. Finally, citations in the identified publications were checked for any omissions in the dataset, and any of these were included by application of the criteria listed above.

## 3. Results

A total of 36 publications were identified using the criteria of inclusion and exclusion stated in the previous section. These included 34 publications primarily describing findings on Symbioflor* E. coli* or the genotypes therein; 2 papers dealt with more than one SymbioPharm product but included Symbioflor* E. coli.* The main findings from these 36 publications are briefly presented here in chronological order.

The oldest references to Symbioflor* E. coli* in the international scientific literature that fulfilled all inclusion criteria that could be identified were from the mid-1990s (older publications on clinical trials and mechanisms exist, but these did not fulfill all inclusion criteria so they were excluded). The deliberate administration of live* E. coli* bacteria to people was not generally accepted in those days. In 1995 Beutin and colleagues reported the result of an expert poll of which the general outcome was the advice that* E. coli *should not be administered for probiotic purposes [2]. After a short introduction by Marget, five experts expressed their concerns. Beutin asked for scientific publications on strain characterization of probiotics. Seeliger would like to know if probiotic* E. coli* actually colonises the human gut in significant numbers. Winberg pointed out that the natural transfer of bacteria from mother to newborn is disturbed by hospital postnatal practices anyway. van der Waaij commented that the biological effect of probiotics on the immune system is dose-dependent and may have opposite effects in low and high doses; he asked for human and animal studies to investigate these effects. Finally, Nord demanded a strain characterization and evidence of colonisation before taking a standpoint. In his conclusion, Marget stated that scientific evidence was needed before probiotic* E. coli *could be considered safe and effective [2].

In the same issue of the journal publishing this “verdict,” Knothe described how serotypes of* E. coli* come and go in a human gut, which in his view would not support the “usefulness of implantation” of a particular, beneficial* E. coli* serovar [3]. Without mentioning Symbioflor* E. coli* directly, his contribution indirectly questioned the use of* E. coli* as a probiotic, based on the assumption that the bacteria would colonise only transiently and when depleted would be easily replaced by other types via natural processes. That other, well-accepted probiotic products based on, for example,* Lactobacillus *spp. or* Bifidobacterium *spp. often also do not colonise the human gut persistently and come and go by natural processes; this was ignored in his discussion. Moreover, it was not known at that time how long or short probiotic strains of* E. coli *actually persist in the gut. The publication by Beutin and colleagues resulted in two published comments, one providing a balanced view of how virulence of a given bacterial strain varies with the host's “resistance of infection” [4] and one pointing out the need to distinguish between pathogenic and nonpathogenic strains within a species, which in their opinion could be done by extensive characterization, where they mentioned* E. coli *Nissle 1917 as an example [5].

Two years later, a very critical review was published in German, asking for scientific evidence of what in those days was called “microbial therapy,” evidence that, in their view, was still lacking [6]. The authors criticised the fact that the original mechanistic explanation for probiotic effects as a means to correct a microbiotic imbalance (“*Symbioselenkung*” to treat “*Dysbacteria*”) had shifted to an immune-modulation effect, as if it were a sin to change a scientific hypothesis in accordance to available data. The authors disagreed with the view that an unbalanced gut flora could be caused by an immunological disorder. Although this had not been extensively shown in those days, it was a working hypothesis for which subsequently an overwhelming amount of evidence has been obtained, albeit mostly from animal studies. The criticism was mainly based on information taken from press releases and commercials and was not directed towards scientific findings [6].

The quest for scientific evidence and a mechanistic explanation of presumed benign actions was started by Jansen and coworkers who posed, in their title, the still provocative question whether* E. coli* could be considered a probiotic [7]. They reported results obtained from 10 healthy volunteers who donated two serum samples (3 weeks apart) prior to a 2-week daily intake of Symbioflor* E. coli*. At the end of the course another serum sample was taken, with a follow-up 4 weeks later. Faecal samples were collected on a weekly basis during the complete investigation. Serum IgG, IgM, and IgA levels were tested for binding capacity to Symbioflor bacteria, whereby the individual's levels prior to intake of the bacteria served as an internal control. Intestinal disturbances following intake of the product were not observed, and the antibody analysis showed an increase in IgG levels only, for all 10 individuals, that lasted throughout the follow-up period [7]. The authors concluded that Symbioflor* E. coli* were probably processed just like any food antigens and doubted an immune-modulation as the result of the product.

In the next publication the effect of Symbioflor* E. coli* on peripheral blood mononuclear cells from healthy human donors was determined* in vitro* and this was compared to the effect of* Enterococcus faecalis*-based Symbioflor 1 as well as the bacterial lysate Pro-Symbioflor (a product containing lysed* E. coli* and* E. faecalis* bacteria) [8]. The response of the mononuclear cells following exposure to these products was compared to the effect of LPS or CpG-containing oligonucleotides. Instead of recording (proinflammatory) cytokine production by means of ELISA, cellular mRNA production was measured quantitatively, as this method was considered more sensitive. Exposure to the three tested products induced increased transcription levels for IL-1b, IL-6, IL-8, MIP1-*α*, MIP2-*α*, and CD69. Although the observed pattern resembled that determined for LPS, these responses could not be due to LPS, since* E. faecalis* (Symbioflor 1) does not contain LPS. Induction of tumor necrosis factor alpha (TNF-*α*) and interferon gamma (IFN-*γ*) as well as interferon-dependent IP-10 was weaker for Pro-Symbioflor than was observed with the live bacteria. Induction of IL-6 and IFN-gamma was particularly high for Symbioflor* E. coli*, where it reached equal or higher levels than the LPS control. Heat treatment abolished the inducing capacity of Symbioflor* E. faecalis* but not that of Symbioflor* E. coli* [8]. The same test system was applied to cell line U-937, derived from myeloid cells, where the products induced transcription of IL-1b, IL-8, TNF-*α*, MIP2-*α*, and MIP1-*α*. For all of these, induction of Symbioflor* E. coli* was the highest, followed by the bacterial lysate, while weak or no induction could be observed for* E. faecalis* [8]. The observed induction patterns were not completely overlapping between U-937 cells and the mononuclear cell preparations, probably because the latter also contained NK-cells, which may have been responsible for the IFN-*γ* response that was absent in U-937 cells.

A very different line of research was described a year later, when Courvoisier and colleagues experimented with detection methods employing fluorescence to detect bacteria in the air or in aerosols in real-time and with high sensitivity. These authors used live* Bacillus subtilis*,* E. coli, *and* E. faecalis* bacteria; for the latter they made use of the Symbioflor strains [9]. Their detection method depends on florescence of the amino acid Tryptophan, which is so robust that it also occurs in living bacteria. They were able to differentiate bacteria from abiotic fluorophores such as diesel fuel. Interestingly, in a follow-up study that no longer used* E. coli*, the authors refined their method and showed that it is applicable to detection of air pollutants [10].

Almost ten years after the trial described by Jansen and coworkers, the bacteria that make up Symbioflor were investigated by microarray analysis, a technique that was en vogue in those days to investigate a bacterial genome before complete DNA sequencing became cheap enough to be generally applied. The used microarray was based on 24* E. coli* and 8* Shigella* genome sequences that were available at that time [11]. Hybridization of the array was validated with DNA from sequenced* E. coli* strains EDL933 (a pathogen) and K-12 MG1655 (a commensal), whose genomes were included in the design for the chip. After those controls, DNA of four of the genotypes present in Symbioflor* E. coli* was analysed, named G1/2, G3/10, G4/9, and G5 (G6/7 and G8 which are also present in the product and closely resemble G1/2 were omitted). This resulted in the first detailed insights about the genetic makeup of these types. The number of predicted genes varied from 3568 (in G4/9) to 3978 (in G1/2), and the four genotypes shared marginally more genes with K-12 MG 1655 than with EDL933. A hierarchical cluster analysis of the hybridization signals also suggested that the genomes of the Symbioflor components more closely resembled* E. coli* K12 than EDL933 or other pathogenic strains. A core genome of 3083 genes was identified that all four genotypes shared [11]. The authors further reported presence of a haemolysin operon (*hlyABCD*) in genotype G1/2, in accordance to the weakly haemolytic phenotype that can be observed for this isolate.

In the introduction of a publication from 2008 it was described how mast cells act as the policemen of the immune system [12], since these cells are alerted by a wide variety of substances, such as bacteria, viruses, or toxins. Upon an encounter with pathogenic bacteria, mast cells release presynthesized substances to recruit the working cells of the immune system (lymphocytes, dendritic cells), but mast cells must differentiate their response between pathogenic and nonpathogenic bacteria. In response to benign bacteria, they produce IL-15, which tunes down the recruitment of immune cells, but a collection of other genes is up- or downregulated during the process. Murine mast cells (MC) were used in an* in vitro* model to investigate how these cells respond to Symbioflor* E. coli* (the product is incorrectly described as Symbioflor 1 in the materials and methods of the publication) [12]. In their model, preincubation of murine mast cells with Symbioflor* E. coli* stopped the cells from being triggered with a calcium ionosphere or an IgG/allergen combination; the inhibition was concentration dependent on a binary manner, with little effect observed below 15,000 bacteria per MC cell, but no further increase at higher inoculates than 15,000. This effect was not observed with sterile culture supernatant or when paraformaldehyde-killed bacteria were used. In a further experiment, mice were injected intraperitoneally with a suspension of the bacteria, and mast cells were harvested a day later from the peritoneum. These cells were again less responsive to the tested triggers. By varying the time between* i.p. *dosage and MC harvest, it was established that the effect faded over the course of a few days [12]. As a side note, it should be mentioned that the mice did not suffer from the bacterial load and survived the treatment till the end of the experiments. Although not stated in the publication, mast cells recognize pathogen-associated molecular patterns (PAMPs) through their pattern recognition receptors (PRRs) such as Toll-like receptors (TLR-4) that are abundant in the mast cell's membrane. Rodent mast cells recognize Gram-negative bacteria via their TLR-4. How exactly Symbioflor* E. coli *is able to inhibit mast cells is currently not known.

A serious attempt for a mechanistic explanation of beneficial effects of Symbioflor treatment was made by Möndel and coworkers, who described that these* E. coli* bacteria induce production of epithelial *β*-defensins in the human host [13]. Such an induction of this defensin had already been shown by* in vitro *studies using* E. coli* Nissle 1917, but* in vivo* data were lacking. After 23 healthy volunteers had taken Symbioflor* E. coli* for 3 weeks, their stool samples contained elevated levels of human *β*-defensin-2 (hBD-2), as determined by ELISA. Such an elevation was not seen with 5 volunteers taking placebo; whether the study was randomised or blinded was not described [13]. The results were confirmed with* in vitro* investigations, whereby three genotypes of Symbioflor were used (here called G1, G2, and G3, corresponding with G1/2, G3/10, and G4/9, resp.) of which only G2 (i.e., G3/10) could induce hBD-2. Interestingly, all three genotypes were equally sensitive to the killing activity of human defensins. The authors speculated that the symbiotic bacteria would require constant administration in order to maintain a stable population in the gut [13].

Reissbrodt and colleagues, on the other hand, investigated the mechanism of action of probiotic bacteria that Alfred Nissle had originally stated for his* E. coli*, namely, the ability to inhibit the growth of pathogenic species, at least* in vitro. *The authors compared Symbioflor* E. coli* to the strain Nissle 1917 and a number of commensal isolates for their ability to inhibit growth of Shiga toxin producing* E. coli* (STEC) [14]. In comparison to* E. coli* Nissle 1917 and a serendipitously found commensal strain, which both reduced Shiga toxin levels with over 90% during cocultivation, the reduction of Shiga toxin was 10 times weaker for Symbioflor* E. coli*, while other* E. coli* strains and Symbioflor 1* E. faecalis* bacteria had no effect at all [14].

In the meantime, the use of probiotic bacteria, including* E. coli*, for treatment of inflammatory bowel disease (IBD) had drawn an interest. Therefore, a randomised double-blind clinical trial was performed to compare Symbioflor* E. coli* treatment of IBD patients with placebo, resulting in significantly more responders (27 out of 148) than in the control group (7/150) [15]. The probiotic was also tested in 203 children with IBD (age range 4–18 years), who tolerated the treatment well and also showed relief of symptoms, although this was not a blinded or placebo-controlled study [16].

In a study describing the passage of bacteria from mother to child,* E. coli* served as the model organism [17]. During early and late pregnancy, mothers donated stool samples and also that from their children in their first week of age and again at 4, 12, and 24 months.* E. coli* could be isolated from the stools of babies as early as day 1 of life, in which case the strain was almost always identical to that of the mother. All isolated* E. coli* strains were characterized for* mdh*, one of the alleles typically used in multilocus sequence typing (MLST). A total of 27 unique strains were thus identified, which were divided (in decreasing order of abundance) into the B2, D, B1, A, and E phylogroups based on related ECOR profiles. By comparing strains found in mother-child combinations it was shown that the percentage of identical strains between them decreased linearly in the first seven days of life [17]. The full MLST genotype was obtained from a subset of isolates and these were compared to probiotic and pathogenic* E. coli *strains; Symbioflor and Nissle 1917 were reported to belong to group B1, while the naturally occurring strains passed from mother to child were predominantly of the B2-type [17]. This study was performed in Norway; it was noted that in other geographical areas, such as in Pakistan, B2 strains were not the main coloniser of infants, and on a global scale group A seems to be predominant.

In a healthy gut, the lumen side of the intestinal epithelium is protected against bacteria by mucus and defensins; this protection is disturbed in IBD, ulcerative colitis (UC), or Crohn's disease (CD) patients. Another possible protective agent is Olfactomedin-4 (OLFM4), a protein that can form large polymeric complexes, similar to mucin. Its function is not yet clear, but it was hypothesized that it is involved in protecting the gut against bacteria, and a role of the protein in the pathogenesis of chronic inflammation disorders was investigated [18]. Biopsies of UC and CD patients revealed that OLFM4 production was increased compared to noninflamed biopsies, in particular in case of UC. A mucin-producing colon adenocarcinoma cell line was used to assess the effect of bacterial components. Exposure to heat-killed extracts of* E. coli* K12 or Nissle 1917 resulted in elevated OLFM4 expression, but heat-killed Symbioflor extracts had no effect [18]. It remained unclear, however, which bacterial factor is responsible for the upregulation seen with some but not all* E. coli *strains. It is also not clear if upregulation of OLFM4 expression plays a role in probiotic activity at all.

Novel insights on the mechanism of probiotic action were obtained when the discovery of Microcin S was described [19]. This novel member of bacteriocins, toxic compounds that bacterial strains produce to destroy competing bacteria, is only produced by Symbioflor genotype G3/10. It is produced from an operon of 4 genes present on the megaplasmid present in this isolate. Activity of the microcin against an enteropathogenic* E. coli *strain (EPEC) was demonstrated* in vitro* [19]. The authors speculated on the use of this novel bacteriocin as a potential antitumor agent. Since G3/10 only comprises 20% of the Symbioflor content, it may explain the weak anti-STEC activity of the product observed by Reissbrodt et al. [14]; if Microcin S is indeed responsible for this activity, performing the experiments with G3/10 only would probably have produced a stronger effect.

A multitude of different types of bacteriocins exist. In a 2013 review article, Cotter and colleagues described how these proteins and peptides are classified into various schemes, such as proteins undergoing and not undergoing posttranslational modification (a classification often used for bacteriocins produced by* Lactobacillus* species), or a classification based on size (small microcins versus larger colicins, a nomenclature typically used for products of Gram-negative bacteria) [20]. The authors argued that the term bacteriocin should be reserved for peptide antimicrobials, which would exclude ribosomally synthesized antimicrobial proteins. They then divided these on the basis of their biochemical modifications, with class I members undergoing modification and class II members lacking such modification. The latter class was further subdivided into IIa to IIe. Using this scheme, Microcin S of Symbioflor* E. coli* belongs to the class IId of unmodified anacyclamides [20].

As more and more studies described the use of probiotics to treat irritable bowel syndrome, IBS (a broader collection of intestinal disorders of which IBD is a subset), Hungin and coworkers performed a systematic review to assess these therapies in adult patients [21]. The authors had identified 37 relevant publications that in total described 32 different probiotics. The majority of those studies focused on IBS with diarrhoeal symptoms; these included the study published by Enck et al. [15] concerning Symbioflor but not that by Martens et al. [16] since the latter had involved pediatric patients. The results described in [15] were cited in 4 of 16 statements by Hungin et al., namely, nr 1: [the product] help(s) relieve overall symptom burden; nr 4: helps reduce abdominal pain; nr 5: helps reduce bloating/distension; nr 8: helps improve frequency and/or consistency of bowel movements; and nr 12: improvement of symptoms leads to improvement in some aspects of health-related quality of life [21]. Two of the statements concerned a negative outcome (nr 6: probiotics tested to date do not help reduce flatus and nr 8: probiotics tested to date do not reduce diarrhoea) for which the Enck study was not supportive. The authors of this systematic review admitted that there is no clear, simple guidance available for physicians, but “(…) our research confirms that there is positive evidence for the role of probiotics in lower GI problems” [21].

It was less than 20 years after the critical evaluation of* E. coli* as a probiotic [2] that publications on mechanistic explanations as well as functionality were available, and the safety of deliberate intake of* E. coli *was no longer questioned* a priori*. This invited researchers to examine Symbioflor bacteria as a means to transfer bioactive molecules safely inside the human body. For instance, it was tested if the bacteria could be used to express recombinant IL-10, as this would be a nice delivery vehicle to get the interleukin in the gut, where it is required for functionality [22]. Disappointingly, although the autotransporter function of haemolysin A, present in Symbioflor genotype G1/2, seemed a suitable means to get the recombinant molecule across the double membrane, it prevented functionality as the protein did not dimerise after secretion. Expression in a probiotic strain of the yeast* Saccharomyces boulardii* had more success [22].

Becker et al. [23] used heat-inactivated bacteria to investigate the effect on cell differentiation and mucin production in an* in vitro *model. The study concentrated on* E. coli *Nissle 1917, but three genotypes of Symbioflor were also included (called G1, G2, and G3, corresponding to G1/2, G3/10, and G4/9, resp.). Two differentiation marker genes of intestinal cells (Hes1 and Hath1) were downregulated as a result of incubation with* E. coli* Nissle 1917 and K-12, while the authors stated that only Hes1 decreased as a result of G3 (after 3 hr) and G2 (after 12 hr incubation) [23]. Their Figure 1, however, suggests that G2 resulted in stronger downregulation of Hes1 than G3.* In vitro* upregulation of hBD-2 was shown for G2 (i.e., G3/10), Nissle 1917, and K-12 [23]. This would suggest that the increased hBD-2 levels demonstrated in exposed volunteers, that had been observed by Möndel and coworkers [13], were the result of genotype G3/10. Becker and colleagues further showed upregulation of Muc1 by* E. coli* G3/10, Nissle 1917, and K-12, resulting in mucin production. The authors suggested that the observed effects by* E. coli *Nissle 1917 were dependent on flagellin. However, this does not explain the observed effects with the Symbioflor bacteria, since these do not express flagellin.

One would think that the science behind probiotics had finally matured, but in 2013 a highly critical paper was published in which the research conducted on the subject of probiotics was severely criticised [24]. The authors highlighted the difficulties of legislators to decide on definitions of probiotics or assessment of their safety, and the diversity in international safety and regulatory standards that were being employed. They pointed at the lack of an independent organisation to direct and conduct research, criticising the wide variety of products being marketed (and the commercial stakes at play), the fuzzy end goals of clinical trials, and the fact that a limited number of products were tested for a wide variety of conditions [24]. The authors were right about all these points, of course, but science is not following logical, targeted, and centrally directed paths, nor would science make the progress it does when scientists were told what to do by a centralized agency. Moreover, the authors were much less critical about the relationship between probiotic agents and the innate immune system, although a lot of those insights are exclusively based on murine models, with questionable relation to the human host. They even considered the work on Segmented Filamentous Bacteria (SFB) noteworthy to mention in a positive light, which disharmonized with their long complaint about undirected and imprecise research. They concluded that probiotic substances do not need to be alive, as bacterial-derived molecular bioactive compounds might be able to do the job [24]. Time will tell if they were right about this.

A second systematic review on the effects of probiotics was published in 2014. Since it covered an even broader collection of intestinal disorders than the study Hungin and colleagues released a year earlier, a total of 356 relevant publications were identified, of which 81 were assessed qualitatively [25]. The clinical symptoms for which trials were conducted were divided into 9 different disorders, of which IBS was the most frequently studied (21 included studies) followed by antibiotic associated diarrhoea (*n* = 17), infectious diarrhoea including viral gastroenteritis (*n* = 11), and necrotizing enterocolitis (*n* = 10) [25]. Concentrating on UC and IBS here, of 27 analysed studies four were investigated that dealt with probiotic* E. coli*. Of these, 3 had used* E. coli* Nissle 1917, with no difference to placebo for one study on IBS, treatment reported as effective as pharmaceutical in remission of UC in one study, and no significant difference in another study on UC. Unfortunately, neither the Enck trial of 2009 (which was published in German) nor the Martens study of 2010 (which dealt with pediatric patients) was included. Instead, another trial on IBD performed by Enck and coworkers was listed, where Pro-Symbioflor (the product containing lysed bacteria of* E. coli *and* E. faecalis*) had been used [26]. That study is not described here any further as it did not deal with live Symbioflor* E. coli*, but in the systematic review by Vitetta et al. [25] it was recorded that abdominal pain and global symptom scores were significantly reduced in that trial. In their conclusions, these authors also asked for more research, with “clinical trials with robust designs and sharp end-points” [25], the same wish that Caselli and colleagues had expressed [24].

A further systematic review and meta-analysis was published in the same year, this time concentrating on IBS and chronic idiopathic constipation [27]. This time 3216 papers were identified to begin with, of which 73 were retrieved for evaluation. After exclusion of 30 papers for various reasons, 43 trials remained, of which 35 described the use of probiotics. The two trials on* E. coli* by Enck and coworkers [15, 26] were included in the meta-analysis (the first one using Symbioflor* E. coli* and the other one using Pro-Symbioflor); the third trial involving* E. coli *that was included had been performed with Nissle 1917 [27]. The position of the Nissle study on the forest plot was comparable to that of the Enck 2009 trial, in terms of effect on persistence of IBS symptoms, while the Enck 2008 results were amongst the most favourable outcomes (RR 0.5). The text mentions an RR = 0.86, 95% CI 0.79–0.93 for all 418 patients receiving* E. coli,* and while stating that a significant effect was only observed with DSM 17252 (i.e., Symbioflor), the results of that trial were nevertheless combined with the ineffective* E. coli *Nissle 1917 data to produce these numbers, as if the strain content of an* E. coli* probiotic can be considered an insignificant variable [27]. Considering the genotypic and phenotypic diversity within this species, this results in misleading conclusions.

In a special issue on irritable bowel syndrome published in the same year, a review was included on IBS in children [28]. Naturally, the authors cited the trial by Martens and coworkers and summarize their results. In their conclusions the authors recommended the inclusion of probiotics in what they called a biopsychosocial approach to treat pediatric IBS, and they specifically mentioned the commercial products VSL#3 and LGG (both not containing* E. coli*) [28].

When an invasive* E. coli* strain UNC was compared to* E. coli *Nissle 1917 in a monoassociation mouse model for mild intestinal inflammation, only strain UNC produced an inflammatory response in IL-10 knockout mice [29]. The invasive UNC bacteria responded to the gut environment by downregulation of glycolytic enzymes, while for the Nissle 1917 strain upregulation of* ivy* was observed, which encodes an inhibitor of the host's lysozyme [29].* In vitro* experiments showed that this overexpression of* ivy* indeed resulted in higher resistance to lysozyme. The* ivy* expression levels of Symbioflor* E. coli *were also determined. After colonising the mice, genotype G1/2 expressed* ivy* at levels similar to those of Nissle 1917, while genotypes 3/10 and G4/9 showed no increase in* ivy* expression. However, the observed difference in* ivy* expression did not correlate with lysozyme resistance in the Symbioflor isolates. Based on sequence analysis, the authors held a naturally mutated promoter in front of the* ivy* gene of* E. coli* Nissle 1917 responsible for its behaviour [29] and were able to show this by overexpression of* ivy*. However, they failed to explain why Symbioflor G1/2 behaved in a similar manner to* E. coli *Nissle and the other genotypes did not; aberrant promoter sequences are not found in these strains. The authors speculated that other, unidentified lysozyme inhibitors might exist that were responsible for the observed effects. As such, the relevance of* ivy* upregulation in mild intestinal inflammation remains uncertain.

One of the still outstanding questions from the original expert opinion publication [2] that had not yet been addressed was dealt with in a 2014 publication in which this author was involved. It described five volunteers who took a single, high dose of Symbioflor* E. coli*, in an attempt to determine how well the bacteria could survive in the gut [30]. It turned out that the bacteria did this surprisingly well: all volunteers were colonised by Symbioflor* E. coli *for at least 12 weeks (27 and 28 weeks in two persons), as specific detection in their stool samples showed. However, after the first week following the single dose intake, all but one genotype of the product had mostly disappeared, while in all volunteers genotype G1/2, combined with G6/7 and G8, survived. This was established with the use of genotype-specific probes that had been designed with the help of the genome sequences. The probes could distinguish between the various genotypes, though G1/2, G6/7, and G8 could not be differentiated [30]. Not all volunteers took the same dose, and by comparison of the highest and lowest dose applied, which were a factor of 100 apart, it was deduced that the persistence of these three genotypes (which together comprise 40% of the product) was not the result of a numerical advantage dictated by the dose. With these findings it was finally established that Symbioflor bacteria are indeed able to colonise the human intestinal tract for multiple weeks, even after a single dose, without causing any side effects. One after another, the concerns expressed in 1995 by Beutin and colleagues [2] were proven irrelevant.

An* in vitro* model was developed to mimic the human gut during onset of an immunological response; the model was used to demonstrate that EDTA releases the epithelial layer from healthy epithelial cells, resulting in activation of immune lamina propria cells. In that way immune cell migration as well as transcriptional responses could be studied. The model was employed to study the effect of bacteria, including Symbioflor [31]. After standardization, drugs such as dexamethasone were tested, which inhibited emigration of lymphocytes in a concentration-dependent manner. Symbioflor* E. coli *was used as an example of nonpathogenic bacteria, which resulted in an increase of inflammatory mediators, in particular IL-1*α* [31].

The genome sequences of the Symbioflor genotypes were made public in 2015, as announced in a publication, with the chromosome of genotype G3/10 sequenced to completion, and the other genotypes (G1/2, G4/9, G5, G6/7, and G8) published as multiple contigs [32]. The plasmids present in these genes were also completely sequenced, and all sequences were made publically available in GenBank. This will enable other research groups to use and employ the genome sequences of Symbioflor* E. coli.*

The finding that there were a number of virulence genes present in the genomes of probiotic Symbioflor, as outlined by Wassenaar and colleagues [33], was no longer a surprise, as by now there was ample evidence that virulence genes are not exclusively found in pathogenic bacteria. The results of the volunteer study were also incorporated in this paper, as well as a ten-year long collation of all side effects collected from commercial use [33]. In view of the large number of sold doses, this list of collected side effects was surprisingly short. Symbioflor* E. coli *was considered to be safe for human consumption, objecting the last concern by Beutin and colleague experts. These findings, though not surprising, make it more difficult to predict the virulent properties of a given* E. coli* strain, even when a complete genome sequence is available, as was discussed in a short paper by Wassenaar and Gunzer [34]. In their publication the authors also drew attention to the genomes of Symbioflor* E. coli *as these contain virulence genes and are nevertheless safe for human use.

The publication by Didari and coworkers focused once more on IBS, with an updated systematic review and meta-analysis [35]. Their analysis started with 170 trials, of which 24 were included based on their eligible criteria. For a meta-analysis, all patients were collectively analysed, which excluded 2 crossover studies, the children's study by Martens, and 6 other trials. Thus only 15 studies remained to be compared, and patients were combined (as is the aim in meta-analyses) when possible. This resulted in an analysis where the 264 patients of Enck et al. 2008 and the 298 patients from Enck et al. 2009 were combined [35], ignoring the fact that in the latter trial Symbioflor was used while in the first Pro-Symbioflor had been applied (the bacterial lysate). In part this mistake may have been caused by unclear descriptions of the products in the original trials, but it also illustrates how difficult it is to perform meta-analyses in this subject, as probiotic products are so heterogeneous.

Mazurak and coworkers also conducted a systematic review, recognizing the weaknesses of the reviews reported here, and for that matter many others were omitted in this summary as they did not include results on Symbioflor [36]. In their systematic review (of which Enck was one of the authors), the study by Enck that used bacterial lysate was omitted, as it was not considered probiotic in the pure sense. Why they also omitted the 2008 study by Enck and coworkers is unclear. The authors recognized the wide diversity in products, applied doses, and treatment duration that limited the possibility of conducting a meta-analysis. They pointed out that over the years the heterogeneity in studies had increased instead of decreasing towards consensus and that few trials followed the rules and proper practice of randomised clinical trials (RCT) as outlined by the FDA, EMA, and the Rome group [36]. In particular, very few trials were registered prior to their start, which is considered an essential step to prevent omission of unwanted data in the final analysis. Many studies were performed with patient numbers being too small to generate the required statistical power to overcome placebo effects. Crossover studies were discouraged by the authors, and they recommended that the EMA and FDA guidelines for clinical trials should always be followed. Lastly, they recommended trials using single-strain probiotics only, thus avoiding the use of products containing multiple strains [36]. This advise would have been clearer if they had advised against the use of mixed species in a product, as that is probably what the authors meant.

Visceral pain (“bellyache” that is difficult to localize) relates to the gut microbiome, as the intestinal bacteria interact with the pathways mediating the sensing of pain. These complex interactions were reviewed by [37]. Most of the insights presented on the interactions along the “microbiota-gut-brain axis” stem from murine models. The human data reviewed in the paper mostly concerned pain in IBS patients, who often have an altered profile of gut microbiota composition, with consistent decrease of* Bifidobacterium* and* Lactobacillus* species, and a general increased ratio of* Firmicutes* to* Bacteroidetes* compared to healthy individuals [37]. That Symbioflor treatment resulting in reduced pain in IBS patients in the Enck trial was mentioned, and although the authors commented that a mechanistic explanation is lacking, increased butyrate-producing bacteria as a result of the probiotic* E. coli* (butyrate reduces rectal pain perception in healthy individuals) were proposed. Alternatively, increased H_2_S production by IBS-microbiota could be the explanation for the beneficial effect [37]. It has not been demonstrated, though, that Symbioflor* E. coli* alter the gut flora in a way that reduces H_2_S production or increases butyrate-producing bacteria.

## 4. Conclusions

From the presented overview of Symbioflor* E. coli* research, which to the knowledge of the author covers all available peer-reviewed literature on this product, it is obvious that the research conducted is neither complete nor unbiased. There has not been a centralized or orchestrated approach to investigate the product, its properties, and functionality in a systematic manner, so that fundamental questions may still remain unanswered. Instead, research is often driven by fashion (each decade of research suffers from “buzzwords”), the urge to discover novelties (even if such discoveries are sometimes exceptional and do not describe the usual), methodology-driven instead of hypothesis-driven, or the result of serendipity. Examples of all of these were encountered during this literature review. Nevertheless, it is of interest to note how one bacterial strain has been used for microbiological research over the years.

From the first, critical publications expressing the view that this* E. coli *was considered unsuitable as a probiotic, works describing its performance in various clinical trials and investigations to reveal the mechanisms of its probiotic actions slowly paved the path to acceptance. Publications characterizing its properties using* in vitro *and* in vivo *(murine) models have become available, while the complete genome sequences revealed the presence of genes that are commonly known as virulence genes. The more information becomes available, the clearer it becomes that even benign bacteria can share a lot of characteristics with pathogens, to an extent that even true virulence genes can be present. Nevertheless, the fact that Symbioflor* E. coli* became one of the standard bacterial strains that is now used as a nonpathogenic control in a variety of publications illustrates that this strain is finally regarded as safe. Although the mechanistic understanding of probiotics is still incomplete, the literature summary provided here may aid the development of pertinent questions for future research.

## Figures and Tables

**Table 1:** Characteristics of Symbioflor 2® containing live, probiotic *E. coli.*

Commercial product	Symbioflor 2 is a suspension of live* E. coli* bacteria for oral intake. The suspension is sold in glass bottles with a drop-release cap. In Germany it is sold over the counter by pharmacists exclusively, but the product is internationally offered online.

Bacteria	Six *E. coli* genotypes.

Content	1.5 to 4.5 × 10^7^ viable bacteria per mL, 50 mL per product unit.

Recommended daily dose	Adults: 3 times daily 10 drops (2 mL) for 1 week and then 3 times daily 20 drops, to be taken with a meal. This corresponds to a daily dose of 6.8–18 × 10^7^ CFU.
	Maximum duration: 6 months.
	Children: daily 10 drops; infants: daily 5 drops.

Maximum daily dose	18 × 10^7^ CFU (60 drops).

*E. coli* genotypes composition	The suspension contains 20% strain G1/2 (DSM 16441), 20% G3/10 (DSM 16443), 20% G4/9 (DSM 16444), 10% G5 (DSM 16445), 20% G6/7 (DSM 16446), and 10% G8 (DSM 16448).
